# Enhancements in the expressions of genes associated with the immunity, muscle growth, and antioxidant activity of 14 d broilers in response to the *in ovo* injection of the Marek's disease vaccine alone or in conjunction with the *in ovo* and dietary supplemental administration of 25-hydroxycholecalciferol

**DOI:** 10.1016/j.psj.2024.104372

**Published:** 2024-09-30

**Authors:** S.A. Fatemi, A.W. Levy, E.D. Peebles

**Affiliations:** ⁎ Department of Poultry Science, Mississippi State University, MS 39762, USA; † DSM Nutritional Products, Parsippany, NJ, 07054, USA

**Keywords:** 25-hydroxycholecalciferol, gene expression, immunity, *in ovo* injection, Marek's disease vaccine

## Abstract

Influences the Marek's disease vaccine (MDV) alone or combined with the *in ovo* and dietary administration of 25-hydroxycholecalciferol (25OHD_3_) on the expression of genes associated with the breast muscle deposition, adaptive and innate immunity, and antioxidant and vitamin D activities of 14 d-old broilers were investigated. Four *in ovo* treatments were: noninjected; commercial MDV-alone-injected (50 μl); or 50 μl of MDV containing 1.2 (MDV+25OHD_3_-1.2) or 2.4 (MDV+25OHD_3_-2.4) μg of 25OHD_3_. Two dietary treatments were a commercial diet containing 25OHD_3_ (250 IU)/kg of feed (control) or the same diet supplemented with additional 25OHD_3 (_2,760 IU)/kg of feed (Hy-D diet). One bird per pen (48 total) was sampled at 14 d for determination of the expression of genes involved with the muscle deposition (MyoD1, MyoG, Pax3, and Mrf4), immunity (INF-γ, IL-10, IL-8, IL-1β, and TGF-β4), antioxidant capacity (SOD1, SOD2, GSH-P1, GSH-P7, and CAT), and vitamin D activity (VDR, 1α-hydroxylase, and 24-hydroxylase) in the spleen and pectoralis major (P.major) muscle. The treatment differences were considered significant at *P* ≤ 0.05. In the P. major, Mrf4 and MyoG were up-regulated in Hy-D-fed birds. Also, the *in ovo* and dietary 25OHD_3_ sources individually increased SOD2 gene expression in the P. major. In the spleen, the expressions of IL-1β and IL-8 were down-regulated and IL-10 and TGF-β4 gene expressions were up-regulated in Hy-D-fed birds than those commercial-fed broiler. *In ovo* and dietary 25OHD_3_ sources enhanced vitamin D gene (1α-hydroxylase and 24-hydroxylase) activities in the breast and spleen. *In ovo* x dietary treatment interactions were significant for the MyoD1, IL-8, Pax3, TGF-β4 genes of the P. major, in which the combined MDV with 1.2 μg of 25OHD_3_ enhanced their expressions in birds fed the Hy-D diet. In conclusion, both 25OHD_3_ sources promoted the expression of genes associated with immunity and P. major growth. It is recommended that both 25OHD_3_ sources can be used to promote the gene expression of 14-day-old broilers in the spleen and breast muscle when MDV administered *in ovo*.

## INTRODUCTION

In the US, the Marek's disease vaccine (**MDV**) is a major vaccine that has been administered using *in ovo* vaccination in broiler hatcheries (Peebles, 2018). Promising results were observed on the humoral immunity of broiler hatchlings in response to the *in ovo* vaccination of MDV at 18 d of incubation (**doi**; Williams, 2007, 2011). Gimeno et al. (2018) suggested that this improvement could be due to an increase in the expressions of genes linked to a stimulation of the immune response, in which the expression of toll like receptor (**TLR**)-3 and interferon (**IFN**)-γ genes in the spleen and the IFN-ß gene in the bursa were increased in broiler hatchlings that received an amniotic injection of the MDV alone at 18 doi. The MDV is a viral vaccine and TLR-3 is an innate immune receptor that primarily detects viruses (El-Zayat et al., 2019). Furthermore, IFN-ß is involved in the stimulation of humoral immunity, while INF-γ operates as a proinflammatory agent (Karpala et al., 2008, 2012). Furthermore, an increase in INF-γ as a result of the *in ovo* administration of the MDV has been observed to increase the secondary humoral responses of 14-day-old broilers (Gimeno et al., 2015).Thus, these results indicate that the MDV can trigger maturation of the immune system. Besides an immunity-related function of the *in ovo* administration of the MDV, it has shown promising results on the small intestine (**SI**) morphology of broiler hatchlings (Peebles et al., 2019) and their subsequent breast meat yield at 49 d of age (**doa**; Peebles et al., 2017). However, the possible molecular mechanism involved in the above-mentioned improvements is not well-understood.

In chickens, vitamin D metabolites, including cholecalciferol (**D_3_**) and 25-hydroxyvitamin D_3_ (**25OHD_3_**), have been shown to exhibit a wide range of effects including growth (Soares et al., 1995; Yarger et al., 1995; Fatemi, 2016), antioxidant activity (Nong et al., 2023), immunity (Morris and Selvaraj, 2014; Morris et al., 2014; Morris et al., 2015), bone formation and development (Fritts and Waldroup, 2003; Bello et al., 2014), and muscle development (Hutton et al., 2014; Vignale et al., 2015; Fatemi, 2016). More specifically, as compared to D_3_, dietary 25OHD_3_ at 2,760 IU (equivalent to 69 μg) has been shown to increase the breast (Yarger et al., 1995; Vignale et al., 2015) and leg (Fatemi, 2016) meat yield, to decrease inflammation during an infection (Morris and Selvaraj, 2014; Morris et al., 2014; Morris et al., 2015; Shanmugasundaram et al., 2019), and to improve the SI morphology (Chou et al., 2009; Ding et al., 2011) of broilers. Furthermore, the *in ovo* injection of 2.4 μg of 25OHD_3_ has been shown to result in an increase in the hatchling quality (Fatemi et al., 2020b), posthatch BW, BW gain (Fatemi et al., 2020a;2021a,b), and breast meat yield (Fatemi et al., 2021b), to decrease the inflammation (Fatemi 2021a,c) and FCR (Fatemi et al., 2020a;2021a,b), and improve the SI morphology (Fatemi et al., 2021c) of Ross 708 broilers under commercial conditions. Additionally, the beneficial results observed on the posthatch live performance, breast and leg meat yield (Fatemi et al., 2021d), immunity, and small intestine morphology (Fatemi et al., 2022a) of broilers subjected to a coccidiosis infection have occurred in response to the *in ovo* injection of 2.4 μg of 25OHD_3_. It is suggested that these improvement are not only due to a significant enhancement of physiological changes, but may also be due to the expression of genes linked to immunity and growth. For instance, the expression of genes linked to the activity of vitamin D receptors (**VDR;** Vignale et al., 2015), 1α-hydroxylase and 24-hydroxylase (Fatemi et al, 2022b), and satellite cell proliferation (Hutton, et al., 2014), are associated with an increase in meat yield. Moreover, innate immunity can be modulated by genes belonged to the TLR family (Fatemi et al., 2022b) and the expression of inflammatory cytokines by macrophages (Morris and Selvaraj, 2014; Shojadoost et al., 2015), while adaptive immunity can be mediated by the stimulation of genes linked to humoral immunity (Fatemi et al., 2024a) or those associated with cell mediated immunity (Morris et al., 2015; Shanmugasundaram et al., 2019; Fatemi et al., 2022b).

More recently, *in ovo* injection of the MDV combined with supplementary 25OHD_3_ through *in ovo* or dietary administration has been shown to not only elevate broiler hatchling BW and the expression of genes related to vitamin D activity (Fatemi et al., 2024a), but to also improve their SI morphology (Fatemi et al., 2024c), inflammatory response (Fatemi et al., 2024c), and increase BW (Fatemi et al., 2024b), at 14 d of age (**doa**) when reared on reused litter. Although the individual molecular mechanisms underlying the effects of the MDV and 25OHD_3_ sources on immunity have been previously reported, their aforementioned effects on immunity, muscle deposition, and antioxidant capacity have not be tested when they were jointly administrated via *in ovo* injection. It is hypothesized that the *in* ovo and dietary sources of 25OHD_3_ alone or in combination can promote the expression of genes linked to growth and immunity when they are provided along with the *in ovo* injection of the MDV. Therefore, in this study, the objectives were to determine the effects of different *in ovo* and dietary levels of 25OHD_3_ coupled with the administration of an *in ovo* injection of the MDV on the expression of genes related to the muscle development, vitamin D activity, immunity, and antioxidant capacity in 14-day-old Ross 708 broilers.

## MATERIALS AND METHODS

### Experimental Design and Treatment Layout

This current study was conducted under the approval of the Mississippi State University Institutional Animal Care and Use Committee (protocol # IACUC-20-248). In each of 12 replicate trays belonging to a Chick Master Incubator (Chick Master Incubator Company, Medina, Ohio), 40 Ross 708 broiler hatching eggs were randomly set in each of 4 *in ovo* injection treatments (1,920 total eggs). All incubational parameters were set in accordance with those described by Lindsey et al. (2023), Fatemi et al. (2023b), and (Mousstaaid et al., 2022, 2023). At 18 doi, each egg in all the prespecified *in ovo* injection treatments were injected with a 50 μL solution volume using a Zoetis Inovoject m machine (Zoetis Animal Health, Research Triangle Park, NC). Included among 4 prespecified in ovo treatment groups were noninjected and commercial MDV-alone-injected controls. Two 25OHD_3_*in ovo* injection treatments included commercial MDV that contained either 1.2 (MDV + 25OHD_3_-1.2) or 2.4 (MDV + 25OHD_3_-2.4) μg of 25OHD_3_. The 25OHD_3_solutions that were freshly prepared as described by Fatemi et al. (2023a, 2024a), contained 25OHD_3_ (ROVIMIX Hy-D 1.25%) that was provided by DSM Nutritional Products Inc. (Parsippany, NJ). At hatch, all birds received starter diets from 0 to 14 doa. After hatch, birds were assigned to 1 of 2 dietary treatments. The dietary treatments were either a control commercial diet containing 250 IU of vitamin D_3_ /kg of feed (control), or the control commercial diet, supplemented with an additional 2,760 IU of 25OHD_3_ /kg of feed (Hy-D diet). A Mississippi State University basal corn-soybean diet was used and all diets were formulated according to Ross 708 commercial guidelines (Aviagen, 2015; Fatemi et al., 2024b).

### Gene Expression Analysis

At 14 doa, 1 bird in each of 48 treatment-replicate combination floor pens was randomly selected and necropsied for tissue collection. Whole spleen and 10 to 15 g samples of the medial apex of the P. major were collected and immediately frozen in liquid nitrogen, and were then stored at −80°C. Total RNA extraction and subsequent RNA quantification were performed according to the procedure described by Fatemi et al., 2022b, 2024a). The glyceraldehyde-3-phosphate dehydrogenase (**GAPDH**) gene was considered as the endogenous or “housekeeping” gene. Furthermore, in the spleen, the expressions of 10 target genes associated with the primary and secondary immunity were analyzed. The targeted genes included TLR-3, TLR-7, TLR-21, INF- α, INF-β, INF-γ, interleukins (**IL**)-1ß, 8, and 10, chicken transforming growth factor-beta 4 (**TGF-β4**), and 3 target genes linked to activity of vitamin D that included 1α-hydroxylase, 24-hydroxylase, and VDR. In addition, in the P. major muscle, the expressions of 5 target genes associated with the immune response included INF-γ, IL-1ß, IL-8, IL-10, and TGF-β4, 6 target genes linked to antioxidant capacity that included superoxide dismutase (**SOD**) 1 and 2, glutathione S-transferase (**GSH**)-P1 and P2, and catalase (**CAT**), 4 target genes linked to muscle deposition that included myogenic differentiation proteins 1 (**MyoD1**) and G (**MyoG**), paired box domain 3 (**Pax3**) and myogenic regulatory transcription factor 4 (**Mrf4**), and the 3 above-mentioned target genes associated with vitamin D activity. Primers for all gene sets have been previously reported (Karpala et al., 2012; Gimeno et al., 2015, Tang et al., 2019; Fatemi et al., 2022b) and are currently presented in Table 1. The cDNA formation and real-time PCR amplifications were performed according to the procedure described by Fatemi et al., 2022b. Fold-change differences were calculated as specified by Fatemi et al. (2022b, 2024a).

**Table 1:** Real-time PCR primers and GenBank accession numbers of chicken housekeeping gene, and target genes associated with immunity, muscle deposition, antioxidant capacity, and vitamin D-related activity.

Gene Symbol	Accession No	Type	Orientation	Sequence (5′ to 3′)	Length (nt)	Amplicon Size (bp)	Reference
GAPDH	NM204305	Housekeeping	Forward	GTAAACCATGTAGTTCAGATCGATGA	26	72	Fatemi et al. (2022b)
			Reverse	GCCGTCCTCTCTGGCAAAG	19		
*IFN-α*	XM_046936231.1	Immunity	Forward	GGACATGGCTCCCACACTAC	20	75	Gimeno et al., 2015
			Reverse	TCCAGGATGGTGTCGTTGAAG	21		
*IFN-β*	AY386204	Immunity	Forward	ACAACTTCCTACAGCACAACAACTA	25	61	Gimeno et al. 2015
			Reverse	GCCTGGAGGCGGACATG	17		
*IFN-γ*	AJ001678	Immunity	Forward	GTGAAGAAGGTGAAAGATATCATGGA	26	71	Fatemi et al. (2022b)
			Reverse	GCTTTGCGCTGGATTCTCA	19		
*TLR-3*	EF137861	Immunity	Forward	GCAACACTTCATTGAATAGCCTTGAT	26	103	Karpala et al. (2012)
			Reverse	ATCAGCAGGTACTCCTCGAT	25		
*TLR-7*	EF587763	Immunity	Forward	TCAGAGGTGGCTGCACAC	18	110	Karpala et al. (2012)
			Reverse	CAACAGTGCATTTGACGTCCTT	22		
*TLR-21*	NM001030558	Immunity	Forward	GTGAAGAAGGTGAAAGATATCATGGA	26	131	Karpala et al. (2012)
			Reverse	GCTTTGCGCTGGATTCTCA	19		
*IL-10*	AJ621614	Immunity	Forward	AGCAGATCAAGGAGACGTTC	20	103	Fatemi et al. (2022b)
			Reverse	ATCAGCAGGTACTCCTCGAT	20		
*TGF-β4*	M31160.1	Immunity	Forward	GGGGTCTTCAAGCTGAGCGT	20	119	Fatemi et al. (2022b)
			Reverse	TTGGCAATGCTCTGCATGTC	20		
*CAT*	NM_0,010,31215.1	Antioxidant activity	Forward	GTTGGCGGTAGGAGTCTGGTCT	22	182	Gimeno et al. (2015)
			Reverse	GTGGTCAAGGCATCTGGCTTCTG	23		
*SOD1*	NM_205,064.1	Antioxidant activity	Forward	CTCACTTTAATCCTGAAGGCAAGC	24	226	Tang et al. (2019)
			Reverse	CCAGTTAGTTTGCTCTCATTATCTCC	25		
*SOD2*	NM_204,211.1	Antioxidant activity	Forward	GTGAACAACCTGAACGTTACAGAGG	25	229	Tang et al. (2019)
			Reverse	GTTTCTCCTTGAAGTTTGCGAAGG	24		
*GSH-Px1*	NM_0,012,77853.1	Antioxidant activity	Forward	TCACCATGTTCGAGAAGTGC	20	124	Tang et al. (2019)
			Reverse	ATGTACTGCGGGTTGGTCAT	20		
*GSH-Px7*	NM_0,011,63245.1	Antioxidant activity	Forward	TTGCAATTACAGCACTCCTGCTC	23	149	Tang et al. (2019)
			Reverse	TGCAACGTTGACAACTAACGACA	24		
*MyoD1*	NM_204214.1	Muscle deposition	Forward	GCCGCCGATGACTTCTATGA	20	66	Yin et al. (2014)
			Reverse	CAGGTCCTCGAAGAAGTGCAT	22		
*MyoG*	NM_204184.1	Muscle deposition	Forward	CGTGTGCCACAGCCAATG	18	63	Yin et al. (2014)
			Reverse	CCGCCGGAGAGAGACCTT	18		
*Pax3*	NM_204269.1	Muscle deposition	Forward	GCCTCACCAGCCCCAAA	17	57	Yin et al. (2014)
			Reverse	GGCTCCAGACCTCCAGTCAA	20		
*Mrf4*	NM_001030746.1	Muscle deposition	Forward	GCGCCATCAGCTACATCGA	19	66	Yin et al. (2014)
			Reverse	GCATTTTGTCCTGCTGATCCA	21		
1α-hydroxylase	XM_422077	Vitamin D activity-related	Forward	TCGTGGCAGGAATACAGAGA	20	125	Fatemi et al. (2022b)
			Reverse	ACTGCCACATCTTTGGGTTT	20		
24-hydroxylase	AF019142.1	Vitamin D activity-related	Forward	AAACCCTGGAAAGCCTATCG	20	133	Fatemi et al. (2022b)
			Reverse	CCAGTTTCACCACCTCCTTG	20		
*VDR*	AF011356.1	Vitamin D activity-related	Forward	CGTGAGAAGCAAATTCAGCA	20	157	Fatemi et al. (2022b)
			Reverse	GAGGTCCAGGTTGGAAAACA	20		

### Statistical Analysis

The experimental design was a randomized complete block with each floor pen serving as an experimental unit. There were 6 replicate groups in each *in ovo* and dietary treatment, resulting in a total of 48 *in ovo* × dietary treatment-replicate groups (4 *in ovo* × 2 dietary × 6 replicates). A normality test was employed using PROC UNIVARIATE for the house-keeping gene as well as all targeted genes, and all were determined as being normally distributed. A 2-way ANOVA with a 2 × 4 factorial arrangement of treatments (2 dietary treatments and 4 *in ovo* injection) was performed to test for significant the main and interactive treatment effects. The General Linear Mixed models procedure (PROC GLIMMIX) of SAS 9.4 (SAS Institute, 2013) was performed for all data, with treatment differences considered to be significant at *P* ≤ 0.05. Fisher's protected least significant difference test was used for treatment means separations (Steel and Torrie, 1980).

## RESULTS

### Immunity

The expression results for the spleen and muscle immunity-related genes are shown in Table 2, Table 3, respectively. No significant main or interactive *in ovo* and dietary treatment effects s were observed for the TLR-3, TLR-7, TLR-21, INF-β, and INF-γ genes in the spleen. However, the effects of the *in ovo* and dietary treatments on the INF-α gene in the spleen significantly interacted. More specifically, in broilers that were fed Hy-D diets, the splenic expression INF-α was higher in those that belonged to the MDV+25OHD_3_-1.2 treatment in comparison to those belonging to the noninjected and MDV+25OHD_3_-2.4 treatments. Also, INF-α gene expression in broilers that received the MDV in combination with either 1.2 or 2.4 μg of 25OHD_3_,was higher when they were subsequently fed the Hy-D supplemented diet rather than the unsupplemented commercial diet. Furthermore, in the spleen, IL-1ß and IL8 gene expressions were down-regulated and IL-10 and TGF-β4 gene expressions were up-regulated in birds fed supplemental Hy-D diets rather than unsupplemented commercial diets. In addition, the IL-1ß gene was up-regulated in birds that were injected with 1.2 or 2.4 μg of 25OHD_3_ when compared to those that were in either the MDV-alone-injected or noninjected treatments. The expressions of the IL-10 and TGF-β4 genes were increased in both the MDV+25OHD_3_-1.2 and MDV+25OHD_3_-2.4 treatment groups as compared to the MDV-alone treatment, whereas IL-10 and TGF-β4 splenic gene expression was up-regulated in birds that received the MDV+25OHD_3_-1.2 treatment in comparison to those that belonged to the MDV-alone-injected and noninjected treatment groups (Table 2).

**Table 2:** Effects of the noninjected, Marek`s disease vaccine-(**MDV**, 50 μL)-alone injected, and MDV-injected in combination with 1.2 or 2.4 μg of 25-hydroxycholecalciferol (**25OHD**_**3**_, 50 μL) *in ovo* treatments; and starter commercial diets or diets supplemented with 2,760 IU/kg of 25OHD_3_ on the fold change expression of immune-related genes in the spleen of 14-day-old broilers.

Treatment	n^6^	TLR-3	TLR-7	TLR-21	INF-α	INF-β	INF-γ	IL-1β	IL-8	IL-10	TGF-β4
*In ovo* injection											
Noninjected^1^	12	1.4	1.3	1.9	1.1	0.9	1.1	1.1^a^	1.9	1.1^bc^	1.3^bc^
MDV^2^	12	1.1	1.3	1.8	1.5	1.2	1.2	1.1^a^	1.4	0.9^c^	1.1^c^
MDV + 25OHD_3_-1.2^3^	12	0.7	1.0	1.8	1.6	1.4	1.2	0.5^b^	0.7	1.7^ab^	1.9^ab^
MDV + 25OHD_3_-2.4^4^	12	0.8	1.4	1.2	1.3	1.1	1.4	0.4^b^	0.9	2.1^a^	2.1^a^
SEM		0.30	0.59	5.1	0.23	0.29	0.29	0.20	0.61	0.34	0.31
Diet											
Commercial	24	1.1	1.5	2.0	0.9	1.0	1.2	0.9^a^	1.8^a^	1.1^b^	1.2^b^
Hy-D^5^	24	1.0	1.0	1.4	1.8	1.2	1.2	0.6^b^	0.7^b^	1.8^a^	2.0^a^
SEM		0.21	0.43	0.36	0.17	0.21	0.20	0.14	0.44	0.24	0.22
Diet × *In ovo* injection											
Commercial*Noninjected	6	1.6	1.6	2.4	0.9^d^	1	1.3	1.3	2.7	0.8	1.0
Commercial*MDV	6	0.9	1.0	1.9	1.2^bcd^	1.1	1.1	1.4	2.0	0.8	0.9
Commercial*MDV + 25OHD_3_-1.2	6	0.8	1.1	2.0	0.7^d^	0.8	0.9	0.7	1.1	1.3	1.5
Commercial*MDV + 25OHD_3_-2.4	6	0.9	2.0	1.5	0.9^d^	1.2	1.6	0.4	1.2	1.5	1.5
Hy-D*Noninjected	6	1.2	0.9	1.3	1.3^bcd^	0.8	0.9	0.9	1.2	1.5	1.6
Hy-D*MDV	6	1.3	1.6	1.8	1.8^ab^	1.2	1.3	0.8	0.8	1.0	1.3
Hy-D*MDV + 25OHD_3_-1.2	6	0.6	0.8	1.7	2.4^a^	2.0	1.5	0.3	0.3	2.2	2.4
Hy-D*MDV + 25OHD_3_-2.4	6	0.8	0.8	1.0	1.6^bc^	1.0	1.6	0.5	0.5	2.8	2.7
SEM		0.43	0.77	0.68	0.36	0.42	0.83	0.36	0.65	0.47	0.44
*P*-value											
*In ovo*	0.104	0.945	0.552	0.156	0.457	0.767	0.001	0.219	0.003	0.009	
Diet		0.681	0.256	0.117	<0.001	0.279	0.869	0.042	0.021	0.003	0.001
*In ovo* × Diet	0.691	0.298	0.724	0.039	0.076	0.188	0.455	0.897	0.435	0.618	

a-c Treatment means within the same column within effect with no common superscripts are significantly different (*P* ≤ 0.05).

1 Embryos that were not injected with a solution.

2 Embryos injected with the Marek's disease vaccine (**MDV**; turkey herpesvirus) at 18 d of incubation (**doi**).

3 Embryos injected with the MDV containing 1.2 μg of 25OHD_3_ at 18 doi.

4 Embryos injected with the MDV containing 2.4 μg of 25OHD_3_ at 18 doi.

5 A diet supplemented with 2,760 IU/kg 25OHD_3_ throughout the rearing period.

6 Number of replications per treatment.

**Table 3:** Effects of the noninjected, Marek`s disease vaccine-(**MDV**, 50 μL)-alone injected, and MDV-injected in combination with 1.2 or 2.4 μg of 25-hydroxycholecalciferol (**25OHD**_**3**_, 50 μL) *in ovo* treatments; and starter commercial diets or diets supplemented with 2,760 IU/kg of 25OHD_3_ on the fold change expression of immune-related genes in the breast muscle of 14-d-old broilers.

Treatment	n^6^	INF-γ	IL-1β	IL-8	IL-10	TGF-β4
*In ovo* injection						
Noninjected^1^	12	1.4	1.2^a^	1.3	1.0^b^	0.9
MDV^2^	12	1.9	0.8^a^	1.8	1.1^b^	1.1
MDV + 25OHD_3_-1.2^3^	12	1.5	0.2^b^	1.2	1.8^ab^	1.6
MDV + 25OHD_3_-2.4^4^	12	1.0	0.6^ab^	0.8	2.0^a^	1.3
SEM		0.63	0.34	0.43	0.43	0.39
Diet						
Commercial	24	1.7	1.3^a^	1.4	1.2	1.1
Hy-D^5^	24	1.2	0.2^b^	1.2	1.7	1.3
SEM		0.44	0.24	0.30	0.30	0.27
Diet × *In ovo* injection						
Commercial*Noninjected	6	1.2	2.3	1.8^ab^	0.7	1.2^b^
Commercial*MDV	6	2.7	1.5	2.7^a^	1.5	1.2^b^
Commercial*MDV + 25OHD_3_-1.2	6	1.7	0.3	0.7^bc^	1.3	0.8^b^
Commercial *MDV + 25OHD_3_-2.4	6	1.2	1.2	0.5^c^	1.5	1.5^ab^
Hy-D*Noninjected	6	1.5	0.2	0.8^bc^	1.2	0.6^b^
Hy-D*MDV	6	1.1	0.2	1.0^bc^	0.7	1.1^b^
Hy-D*MDV + 25OHD_3_-1.2	6	1.3	0.1	1.7^ab^^c^	2.7	2.4^a^
Hy-D*MDV + 25OHD_3_-2.4	6	0.7	0.2	1.2^bc^	2.2	1.2^b^
SEM		0.88	0.48	0.60	0.60	0.55
		—————————-*P*-value———————-				
*In ovo*	0.519	0.012	0.166	0.050	0.279	
Diet		0.253	0.002	0.405	0.137	0.518
*In ovo* × Diet	0.503	0.244	0.007	0.067	0.049	

a-c Treatment means within the same column within effect with no common superscripts are significantly different (*P* ≤ 0.05).

1 Embryos that were not injected with a solution.

2 Embryos injected with the Marek's disease vaccine (MDV; turkey herpesvirus) at 18 d of incubation (doi).

3 Embryos injected with the MDV containing 1.2 μg of 25OHD_3_ at 18 doi.

4 Embryos injected with the MDV containing 2.4 μg of 25OHD_3_ at 18 doi.

5 A diet supplemented with 2,760 IU/kg 25OHD_3_ throughout the rearing period.

6 Number of replications per treatments.

In the P. major muscle, there was no significant interaction or main effects between the dietary and *in ovo* injection for INF-γ. However, there were significant interactive effects between the *in ovo* and dietary treatments for the IL-8 and TGF-β4 genes. In the commercial diet, the expression of IL-8 was up-regulated in birds belonging to the MDV-alone-injected treatment in comparison to those belonging to the MDV+25OHD_3_-1.2 and MDV+25OHD_3_-2.4 treatment groups, and it was higher in both control treatment groups (MDV-alone-injected and noninjected) than that in the MDV+25OHD_3_-1.2 treatment. Additionally, IL-8 expression was higher in the MDV-alone-injected birds fed commercial diets rather than those fed Hy-D diets. In birds fed the supplemental Hy-D diets, a higher TGF-β4 expression was observed in those that received the MDV+25OHD_3_-1.2 treatment as compared to those in all the other *in ovo* injection treatments, but there was no significant treatment difference among the *in ovo* injection treatments in those fed the unsupplemented commercial diet. Birds fed supplemented Hy-D diets experienced a down-regulation in the expression of IL-1ß in the P. major muscle compared to those fed unsupplemented commercial diets. Furthermore, the expression of IL-1ß was decreased and TGF-β4 was increased in birds that received the MDV+25OHD_3_-1.2 treatment as compared to those that belonged to the MDV-alone-injected and noninjected control treatment groups (Table 3).

### Antioxidant Activity

The activity results for the antioxidant-related genes are illustrated in Table 4. There were not only no significant interactive effects between the dietary and *in ovo* and treatments for the activities of all the antioxidant-related activity genes. Also, the expression of the GSH-P1, GSH-P7, and CAT genes did not differ in both *in ovo* or dietary treatment main effects. However, the increased expression of SOD2 in the P. major muscle was observed in birds fed supplemented dietary Hy-D diets as compared to those fed unsupplemented commercial diets. In addition, the expressions of SOD1 and SOD2 were increased in birds that belonged to the MDV+25OHD_3_-1.2 treatment as compared to those in the MDV-alone-injected and noninjected control treatment groups. Additionally, the expression of SOD2 was up-regulated in birds that belonged to the MDV+25OHD_3_-2.4 treatment in comparison to those in the MDV-alone-injected and noninjected control treatment groups.

**Table 4:** Effects of the noninjected, Marek`s disease vaccine-(**MDV,** 50 μL)-alone injected, and MDV-injected in combination with 1.2 or 2.4 μg of 25-hydroxycholecalciferol (**25OHD**_**3**_, 50 μL) *in ovo* treatments; and starter commercial diets or diets supplemented with 2,760 IU/kg of 25OHD_3_ on the fold change expression of antioxidant activity-related genes in the breast muscle of 14-d-old broilers.

Treatment	n^6^	SOD1	SOD2	GSH-P1	GSH-P7	CAT
*In ovo* injection						
Noninjected^1^	12	0.8^b^	0.6^b^	1.0	1.3	1.3
MDV^2^	12	1.0^b^	0.9^b^	1.5	0.9	1.1
MDV + 25OHD_3_-1.2^3^	12	1.2^ab^	2.3^a^	2.2	1.4	1.5
MDV + 25OHD_3_-2.4^4^	12	1.6^a^	2.8^a^	1.6	1.5	1.3
SEM		0.24	0.38	0.71	0.5	0.42
Diet						
Commercial	24	1.1	1.2^b^	1.4	1.2	1.3
Hy-D^5^	24	1.2	2.1^a^	1.7	1.3	1.4
SEM		0.17	0.27	0.50	0.27	0.30
*P*-value						
*In ovo*		0.026	<0.001	0.408	0.706	0.700
Diet		0.778	0.003	0.408	0.881	0.834
*In ovo* x Diet	0.278	0.372	0.835	0.407	0.408	

a,b Treatment means within the same column within effect with no common superscripts are significantly different (*P* ≤ 0.05).

1 Embryos that were not injected with a solution.

2 Embryos injected with the Marek's disease vaccine (MDV; turkey herpesvirus) at 18 d of incubation (doi).

3 Embryos injected with the MDV containing 1.2 μg of 25OHD_3_ at 18 doi.

4 Embryos injected with the MDV containing 2.4 μg of 25OHD_3_ at 18 doi.

5 A diet supplemented with 2,760 IU/kg 25OHD_3_ throughout the rearing period.

6 Number of replications per treatment.

### Muscle Deposition

The results for the muscle deposition-related genes are demonstrated in Table 5. The expression of the MyoD1 and Pax3 genes did not affect by interaction effects between the *in ovo* and dietary treatments. In birds fed the Hy-D diet, the expression of MyoD1 was increased in the MDV + 25OHD_3_-1.2 treatment in comparison to that of birds in the MDV-alone-injected and MDV + 25OHD_3_-2.4 *in ovo* injection treatments, and a down-regulation in the expression of MyoD1was observed in the MDV-alone-injected treatment as compared to those belonging to the MDV+25OHD_3_-1.2 treatment. Furthermore, in birds fed the Hy-D diet, those that were in the MDV + 25OHD_3_-1.2 treatment experienced a higher expression of Pax3 as compared to all other *in ovo* injection treatments. Moreover, the expressions of MyoG and MrfF4 were increased in birds fed Hy-D diets in comparison to those fed commercial diets.

**Table 5:** Effects of the noninjected, Marek`s disease vaccine-(**MDV**, 50 μL)-alone injected, and MDV-injected in combination with 1.2 or 2.4 μg of 25-hydroxycholecalciferol (**25OHD**_**3**_, 50 μL) *in ovo* treatments; and starter commercial diets or diets supplemented with 2,760 IU/kg of 25OHD_3_ on the fold change expression of muscle deposition-related genes in the breast muscle of 14-d-old broilers.

Treatment combination	n^6^	MyoD1	MyoG	Pax3	MRF4
*In ovo* injection					
Noninjected^1^	12	1.1	1.0	1.1	1.3
MDV^2^	12	1.0	1.1	1.4	1.1
MDV + 25OHD_3_-1.2^3^	12	2.4	1.6	2.1	1.9
MDV + 25OHD_3_-2.4^4^	12	1.5	1.3	1.6	1.8
SEM		0.25	0.32	0.29	0.47
Diet					
Commercial	24	1.1	0.9^b^	1.0	1.1^b^
Hy-D^5^	24	1.9	1.6^a^	2.1	1.9^a^
SEM		0.18	0.23	0.21	0.33
Diet × *In ovo* injection					
Commercial *Noninjected	6	1.1^bc^	0.7	0.8^d^	1.1
Commercial *MDV	6	0.5^c^	0.7	1.2bcd	1.0
Commercial *MDV + 25OHD_3_-1.2	6	1.5^b^	1.1	1.0^cd^	1.1
Commercial *MDV + 25OHD_3_-2.4	6	1.2^bc^	0.9	1.3bcd	1.1
Hy-D*Noninjected	6	1.2^bc^	1.3	1.4bcd	1.5
Hy-D*MDV	6	1.4^b^	1.4	1.6^bc^	1.1
Hy-D*MDV + 25OHD_3_-1.2	6	3.2^a^	2.1	3.3^a^	2.8
Hy-D*MDV + 25OHD_3_-2.4	6	1.8^b^	1.6	2.0^b^	2.4
Pooled SEM		0.36	0.46	0.41	0.67
*P*-value					
*In ovo*		<0.001	0.227	0.008	0.243
Diet		<0.001	0.003	<0.001	0.012
	*In ovo* x Diet	0.014	0.884	0.013	0.280

a-d Treatment means within the same column within effect with no common superscripts are significantly different (*P* ≤ 0.05).

1 Embryos that were not injected with a solution.

2 Embryos injected with the Marek's disease vaccine (MDV; turkey herpesvirus) at 18 d of incubation (doi).

3 Embryos injected with the MDV containing 1.2 μg of 25OHD_3_ at 18 doi.

4 Embryos injected with the MDV containing 2.4 μg of 25OHD_3_ at 18 doi.

5 A diet supplemented with 2,760 IU/kg 25OHD_3_ throughout the rearing period.

6 Number of replications per treatment.

### Vitamin D Activity

The results for the muscle deposition-related genes are exhibited in Table 6. All the vitamin D activity-related genes in both the spleen and P. major muscle did not affect by the interaction effect between the *in ovo* and dietary treatments. An up-regulation in the expression of 1α-hydroxylase and a down-regulation in the expression of 24-hydroxylase were observed in the spleen and P. muscle of broilers fed supplemental dietary Hy-D in comparison to those fed unsupplemented commercial diets. Furthermore, the splenic expression of 1α-hydroxylase was increased in birds that received the MDV+25OHD_3_-1.2 treatment in comparison to those in the MDV-alone-injected and noninjected treatment groups. Moreover, a splenic up-regulation of 24-hydroxylase occurred in birds in the MDV-alone-injected and noninjected treatment groups in comparison to those belonging to the MDV+25OHD_3_-1.2 and MDV+25OHD_3_-2.4 treatment groups.

**Table 6:** Effects of the noninjected, Marek`s disease vaccine-(**MDV**, 50 μL)-alone injected, and MDV-injected in combination with 1.2 or 2.4 μg of 25-hydroxycholecalciferol **(25OHD**_**3**_, 50 μL) *in ovo* treatments; and starter commercial diets or diets supplemented with 2,760 IU/kg of 25OHD_3_ on the fold change expression of vitamin D activity-related genes in the breast muscle and spleen of 14-day-old broilers.

Treatment	n^6^	1α-hydroxylase	24-hydroxylase	VDR
		Breast muscle		
*In ovo* injection				
Noninjected^1^	12	0.5^b^	2.4	2.0
MDV^2^	12	1.0^b^	1.7	2.0
MDV + 25OHD_3_-1.2^3^	12	1.4^ab^	1.4	2.1
MDV + 25OHD_3_-2.4^4^	12	1.7^a^	1.2	1.8
SEM		0.35	0.50	0.79
Diet				
Commercial	24	0.9^b^	2.1^a^	1.7
Hy-D^5^	24	1.4^a^	1.3^b^	2.2
SEM		0.25	0.35	0.56
		*P*-value		
*In ovo*		0.013	0.087	0.992
Diet		0.043	0.037	0.413
In ovo x Diet	0.130	0.702	0.827	
		Spleen		
*In ovo* injection				
Noninjected^1^	12	1.1	2.0^a^	1.4
MDV^2^	12	1.2	1.8^a^	1.5
MDV + 25OHD_3_-1.2^3^	12	2.1	0.9^b^	2.1
MDV + 25OHD_3_-2.4^4^	12	1.8	0.8^b^	1.4
SEM		0.63	0.30	0.71
Diet				
Commercial	24	1.1^b^	1.7^a^	1.7
Hy-D^5^	24	2.1^a^	1.0^b^	1.5
SEM		0.38	0.21	0.50
*In ovo*		*P*-value		
Diet		0.318	0.001	0.692
*In ovo* x Diet	0.027	0.002	0.639	
*In ovo* x Diet	0.833	0.147	0.876	

a-d Treatment means within the same column within effect with no common superscripts are significantly different (*P* ≤ 0.05).

1 Embryos that were not injected with a solution.

2 Embryos injected with the Marek's disease vaccine (MDV; turkey herpesvirus) at 18 d of incubation (doi).

3 Embryos injected with the MDV containing 1.2 μg of 25OHD_3_ at 18 doi.

4 Embryos injected with the MDV containing 2.4 μg of 25OHD_3_ at 18 doi.

5 A diet supplemented with 2,760 IU/kg 25OHD_3_ throughout the rearing period.

6 Number of replications per treatment.

## DISCUSSION

The influences of different *in ovo-*injected and dietary 25OHD_3_ doses in conjunction with the *in ovo* administration of the MDV on the gene expression of 14-d-old broilers raised under commercial conditions were investigated in this study. The genes in this study were associated with the innate immune response (TLR-3, TLR-7, and TLR-21) as well as adaptive immune responses. The genes associated with adaptive immunity included those that were specifically involved with humoral immunity (INF-α and INF-β), and proinflammatory (INF-γ, IL-1ß, and IL-8) and anti-inflammatory (IL-10 and TGF-β4) responses. Those genes active in humoral and innate immune responses were only tested in the spleen, which is the primary immune tissue for antibody production. The results of this study indicated that INF-α expression in birds fed Hy-D diets and that received the MDV, was elevated in response to the *in ovo* injection of 25OHD_3_ across both doses. Similarly, the expression of INF-α in *in ovo* MDV-injected birds has been previously shown to increase in response to the *in ovo* injection of 25OHD_3_ when compared to those that were not *in ovo*-injected with 25OHD_3_ (Fatemi et al., 2024a). It is well known that an increase in the expression of INF-α results in a stimulation of antibody production in the primary immune tissues of chickens (Lyton et al., 2022) and mammals (Zhang et al., 2017). Thus, these results indicate that both sources of 25OHD_3_ administrated in combination with the *in ovo* injection of the MDV have the potential to influence the expression of INF-α, which has the potential to stimulate humoral immunity. The expressions of IL-1ß, and IL-8, which are considered as proinflammatory cytokines, were down-regulated in the spleen in response to the dietary administration of 25OHD_3_ in this study. In addition, the expressions of IL-10 and TGF-β4, which are involved in anti-inflammatory reactions, were up-regulated in the spleens of the broilers fed supplemental Hy-D diets. The expressions of the IL-1ß and IL-8 genes are linked to an increases in inflammatory responses that result in an increase immune reactions that can be beneficial if host cells experience a pathogenic invasion (Morris and Selvaraj, 2014; Morris et al., 2014; Shojadoost et al., 2015). This would result in an increase in inflammatory responses involving a wide range of immune cells including, B- and T-cells (Lowenthal et al., 2000; Wigley and Kaiser, 2003). An increased immune response would be beneficial during pathogenic invasion or immediately after vaccination; however, a chronically stimulated immune reaction has been shown to have an adverse effect on growth (Morris and Selvaraj, 2014; Morris et al., 2015; Shanmugasundaram et al., 2019; Fatemi et al., 2021a, 2022a). Furthermore, under commercial conditions, an increase in anti-inflammatory responses are highly linked to an increase in growth (Morris and Selvaraj, 2014; Morris et al., 2015; Shanmugasundaram et al., 2019) and breast meat yield (Fatemi et al., 2021c, Fatemi et al., 2021d; Fatemi et al., 2022a, Fatemi et al., 2022b) in broilers. Both IL-10 and TGF-β4 trigger regulatory T cell (**Tregs**) formation that can result in the suppression of immunity cells by inhibiting T cell proliferation and cytokine production (Wan and Richard, 2007; Sojka et al., 2008; Shanmugasundaram and Selvaraj, 2011). In this study, both the dietary and *in ovo* 25OHD_3_ sources showed promising results in the up-regulation of anti-inflammatory response genes in either the spleen or breast muscle. In the previous companion study (Fatemi et al., 2024c), the serum concentrations of α 1-acid glycoprotein (**AGP**), known as a chronic inflammatory indicator, were decreased at 14 doa in broilers fed supplemental dietary 25OHD_3_ and which had previously received the MDV by *in ovo* injection. Additionally, in comparison to noninjected and diluent-injected control treatment groups, the *in ovo* injection of 25OHD_3_ has been shown to reduce AGP concentrations in broilers (Fatemi et al., 2021a). Therefore, reductions in systemic inflammation as a result of the *in ovo* or dietary use of supplemental 25OHD_3_ could be partially linked to an up-regulation of the anti-inflammatory and down-regulation of the proinflammatory response genes.

It is well-documented that woody breast myopathy (**WBM**), which is an abnormality in the P. major leading to hard and thick breast meat, is due to an increase in inflammation in connective tissue and oxidative stress in the breast fillet (Kuttappan et al., 2013; Sihvo et al., 2014). In addition, the rate of fat deposition has been observed to increase and protein synthesis to decrease in the breast fillets of broilers exhibiting WBM (Kuttappan et al., 2013; Trocino et al., 2015). Woody breast myopathy is mainly controlled by environmental factors including diet and management rather than genetic selection (Bailey et al., 2015). Additionally, the development of WBM has been shown to initiate in newly hatched chicks (Kuttappan et al., 2013; Caldas-Cueva and Owens, 2020). Although WBM incidence is not visible in younger birds, the morphological changes in breast filet have been detected in early post-hatch chicks. In this study, among the 5 enzymatic antioxidant-activity genes investigated, SOD1 and SOD2 were up-regulated by administration of the *in ovo* and dietary sources of 25OHD_3_. These genes are involved in the production of the superoxide dismutase enzyme, which belongs to the first level of the antioxidant defense network. This enzyme provides effective protection against lipid peroxidation in chickens in (Surai et al., 2019). Thus an increase in antioxidant capacity-related genes and anti-inflammatory response genes, and a decrease in proinflammatory response genes, may contribute in lowering the development of WBM in young broilers. However, further research in needed to identify the morphological changes in breast fillets throughout the rearing period in response to both the *in ovo* and dietary sources of 25OHD_3_ in birds having received *in ovo* administration of the MDV.

The patterns of chicken myogenic regulatory factor (**MRF**) expression, including those of MyoG, Mrf4, myogenic factor 5 (**Myf5**), and myogenic differentiation protein (**MyoD**) determine skeletal muscle lineage (Liu et al., 2012; Yablonka-Reuveni and Paterson, 2001). The combined expressions of the Myf5 and MyoD genes are associated with the maintenance of myoblasts (Haldar et al., 2008; Mok et al., 2015), followed by the expression of the MyoG gene, which is involved with the onset of muscle differentiation. The MyoG gene also serves as a differentiation factor in myogenesis, through its contribution towards cell lineage specification during muscle myoblast formation (Moncaut et al., 2013). Furthermore, MyoG and Myf5 can regulate their own expressions while interacting with MyoD. More specifically, MyoD and MyoG play key roles in the differentiation and multiplication of fetal and adult chicken myoblasts (Yablonka–Reuveni and Paterson, 2001). In addition, expressions of the MyoG and Myf5 genes are associated with an increase in breast and leg muscle development during the early post-hatch and adult lives of chickens (Genxi et al., 2014). It is well observed that the active form of vitamin D can directly affect the expression of MRF genes that can result in change in muscle development and subsequently breast meat yield (van der Meijden et al., 2016). This can be facilitated by change in the expression of genes associated with vitamin D activity, in which an increase in 1α-hydroxylase can lead to an increase in 1,25(OH)_2_ D_3_ level and therefore enhanced MRF gene expression or the adverse effect can be expected by up-regulation of 24-hydroxylase leading to inactive form of vitamin D, 24,25(OH)_2_ D_3_.

More recently, it has been shown that the *in ovo* injection of vitamin D sources including 25OHD_3_ at 12 doi results in the up-regulation of MRF genes in broiler embryos (Chen et al., 2021). Additionally, an increased expression of MyoG has been shown to be associated with increased satellite cell (**SC**) proliferation (Clark et al., 2016). The Pax3 gene, which is another MRF gene, is involved in the proliferation of SC-derived myoblasts (Shinin et al., 2006; Day et al., 2007). Hutton et al. (2014) reported that supplemental 25OHD_3_ at 2,670 IU/kg in feed resulted in an increased meat yield and protein synthesis in breast fillets. Therefore, an increased expression of MRF genes can result in increased meat production in chickens (Genxi et al., 2014). Results of the companion study conducted by Fatemi et al. (2024b) revealed that dietary supplemental 25OHD_3_ increased P. major and breast meat yield and that the *in ovo* injection of 2.4 μg of 25OHD_3_ increased the pectoralis minor yield at 14 doa in broilers that had received the MDV by *in ovo* injection. Furthermore, in the same companion study, an enhancement in broiler live performance was observed in response to both *in ovo* and dietary sources of 25OHD_3_. In the current study, an up-regulation in the expression of the MRF genes was observed in birds provided both 25OHD_3_ sources. However, this up-regulation was more prominent in response to use of the dietary source of 25OHD_3_. Therefore, an improvement in the breast meat yield and live performance variables of broilers in response to the dietary or *in ovo* administration of 25OHD_3_ could be partially linked to an up-regulation in the expression of the MRF genes.

Other potential bases for the positive results in the expressions of the immunity- and muscle development-related genes in response to both 25OHD_3_ sources could be linked to an increase in the activity of 1α-hydroxylase and a decrease in the activity of 24-hydroxylase in spleen and muscle tissues. It is well observed that the conversion 25OHD_3_ to the active form of vitamin D, 1,25(OH)_2_ D_3_, is facilitated by 1α-hydroxylase. Furthermore, 25OHD_3_ can be converted to the inactive form of vitamin D, 24,25(OH)_2_ D_3_, by the action of 24-hydroxylase. (Shanmugasundaram and Selvaraj, 2012). Previously, an up-regulation in the anti-inflammatory response genes, IL-10 and TGF-β4, was observed to be associated with an increased activity of 1α-hydroxylase and a decreased activity of 24-hydroxylase (Fatemi et al., 2022b). It is well-documented that an increase in the circulating level of 1,25(OH)_2_ D_3_ is associated with an increased intestinal absorption of Ca and P (De Matos, 2008), an enhancement of immunological activity (Shanmugasundaram and Selvaraj, 2012; Shojadoost et al., 2015; Shanmugasundaram et al., 2019; Fatemi et al., 2021d, 2022b), and increases in the live performance and breast meat yield of broilers (Fatemi et al., 2021d, Fatemi et al., 2022b). Thus, higher concentrations of 1,25(OH)_2_ D_3_ in target tissues can be linked to greater and robust effects on immunological and growth variables. However, an increase in tissue concentrations of 24,25(OH)_2_ D_3_ could result in adverse effects. Therefore, beneficial effects in spleen and breast muscle tissues in response to both 25OHD_3_ sources could be partially linked to an improvement in the expression of vitamin D activity-related genes in those tissues.

In conclusion, the posthatch expression of genes linked to immunity, antioxidant activity, muscle deposition, and vitamin D activity in14-day-old broilers in response to various doses of supplemental *in ovo* and dietary 25OHD_3_ administration in combination with *in ovo* delivery of the MDV were investigated. The findings in the current study revealed that in comparison to an MDV-alone-injected treatment group, both levels of *in ovo-*injected of 25OHD_3_ were effective in up-regulating the expression of the IL-10 and1α-hydroxylase genes in the breast muscle and spleen tissues. Those treatments were likewise instrumental in the increased expression of the SOD2 gene in the breast muscle, and down-regulated expressions of the genes for IL-1β in the spleen and IL-8 in the breast muscle of broilers fed diets supplemented with Hy-D. In addition, increased expressions of the MyoD1 and Pax3 genes were observed in birds fed Hy-D diets and that belonged to the the MDV + 25OHD_3_-1.2 treatment. Furthermore, increased expressions of the immunity-related (IL-1β, IL-8, IL-10, and TGF-β4) and vitamin D activity-related (1α-hydroxylase and 24-hydroxylase) genes in both the spleen and muscle, and the antioxidant capacity-related (SOD1 and SOD2) and muscle deposition-related (Mrf4 and MyoG) genes in the breast muscle were observed in birds fed supplemental Hy-D rather than un-supplemental commercial diets. The promising results in response to 25OHD_3_ sources could be linked to an enhancement of genes associated with increased expression of active form vitamin D. These results indicate that both 25OHD_3_ sources are capable of functionally improving the expressions of genes that are associated with immunity and growth. However, the dietary source of 25OHD_3_ appeared to be a more potent stimulant than the *in ovo* source. The positive results in response to the 25OHD_3_ sources may be due to an enhancement of genes associated with vitamin D activity in both the spleen and breast muscle. Therefore, it is suggested that the supplemental administration of 25OHD_3_ via *in ovo* injection or the diet can be used to trigger the expression of genes involved in immunity and muscle deposition in broilers that are *in ovo*-injected with the MDV.

## DISCLOSURES

There is no conflict of interest.

## References

[bib0001] Aviagen (2015). http://en.aviagen.com/assets/Tech_Center/BB_Resources_Tools/Pocket_Guides/Ross-Broiler-Pocket-Guide-2015-EN.pdf.

[bib0002] Bailey R.A., Watson K.A., Bilgili S.F., Avendano S. (2015). The genetic basis of pectoralis major myopathies in modern broiler chicken lines. Poult. Sci..

[bib0003] Bello A., Hester P.Y., Gerard P.D., Zhai W., Peebles E.D. (2014). Effects of commercial *in ovo* injection of 25-hydroxycholecalciferol on bone development and mineralization in male and female broilers. Poult. Sci..

[bib0004] Caldas-Cueva J.P., Owens C.M. (2020). A review on the woody breast condition, detection methods, and product utilization in the contemporary poultry industry. J. Anim. Sci.

[bib0005] Chen C., White D.L., Marshall B., Kim W.K. (2021). Role of 25-Hydroxyvitamin D_3_ and 1,25-Dihydroxyvitamin D_3_ in chicken embryo osteogenesis, adipogenesis, myogenesis, and vitamin D_3_ metabolism. Front. Physiol..

[bib0006] Chou S.H., Chung T.K., Yu B. (2009). Effects of supplemental 25-hydroxycholecalciferol on growth performance, small intestinal morphology, and immune response of broiler chickens. Poult. Sci..

[bib0007] Clark D.L., Coy C.S., Strasburg G.M., Reed K.M., Velleman S.G. (2016). Temperature effect on proliferation and differentiation of satellite cells from turkeys with different growth rates. Poult. Sci..

[bib0008] Day K., Shefer G., Richardson J.B., Enikolopov G., Yablonka-Reuveni Z. (2007). Nestin-GFP reporter expression defines the quiescent state of skeletal muscle satellite cells. Dev. Biol.

[bib0009] De Matos R. (2008). Calcium metabolism in birds. Vet. Clin. North. Am. Exot. Anim. Pract..

[bib0010] Ding B.A., Pirone A., Lenzi C., Baglini A., Romboli I. (2011). Effect of hen diet supplemented with 25-OH-D_3_ on the development of small intestinal morphology of chick. J. Anim. Feed. Sci..

[bib71] El-Zayat S.R., Sibaii H., Mannaa F.A. (2019). Toll-like receptors activation, signaling, and targeting: an overview. Bull. Natl. Res. Cent..

[bib0011] Fatemi S.A. (2016).

[bib0012] Fatemi S.A., Elliott K.E.C., Bello A., Durojaye O., Zhang H., Turner B., Peebles E.D. (2020). The effects of *in ovo* injected vitamin D_3_ sources on the eggshell temperature and early posthatch performance of Ross 708 broilers. Poult. Sci..

[bib0013] Fatemi S.A., Elliott K.E.C., Bello A., Durojaye O., Zhang H., Turner B., Peebles E.D. (2020). Effects of source and level of *in ovo*-injected vitamin D_3_ on the hatchability and serum 25-hydroxycholecalciferol concentrations of Ross 708 broilers. Poult. Sci..

[bib0014] Fatemi S.A., Alqhtani A., Elliott K.E.C., Bello A., Zhang H., Levy A.W., Peebles E.D. (2021). Improvement in the performance and inflammatory reaction of Ross 708 broilers in response to the *in ovo* injection of 25-hydroxyvitamin D_3_. Poult. Sci..

[bib0015] Fatemi S.A., Elliott K.E.C., Bello A., Zhang H., Alqhtani A.H., Peebles E.D. (2021). Effects of the *in ovo* injection of vitamin D_3_ and 25-hydroxyvitamin D_3_ in Ross 708 broilers subsequently fed commercial or calcium and phosphorous-restricted diets: I. performance, carcass characteristics, and incidence of woody breast myopathy. Poult. Sci..

[bib0016] Fatemi S.A., Elliott K.E.C., Bello A., Zhang H., Peebles E.D. (2021). Effects of the *in ovo* injection of vitamin D_3_ and 25-hydroxyvitamin D_3_ in Ross 708 broilers subsequently fed commercial or calcium and phosphorous-restricted diets: II. Immunity and small intestine morphology. Poult. Sci..

[bib0017] Fatemi S.A., Elliott K.E.C., Bello A., Peebles E.D. (2021). Effects of the *in ovo* injection of vitamin D_3_ and 25-hydroxyvitamin D_3_ in Ross 708 broilers subsequently challenged with coccidiosis. I. performance, meat yield and intestinal lesion. Poult. Sci..

[bib0018] Fatemi S.A., Elliott K.E.C., Macklin K.S., Bello A., Peebles E.D. (2022). Effects of the *in ovo* injection of vitamin D_3_ and 25-hydroxyvitamin D_3_ in Ross 708 broilers subsequently challenged with coccidiosis: II immunological and inflammatory responses and small intestine histomorphology. Animals (Basel).

[bib0019] Fatemi S.A., Macklin K.S., Zhang L., Mousstaaid A., Poudel S., Poudel I., Peebles E.D. (2022). Improvement in the immunity- and vitamin D_3_ activity-related gene expression of coccidiosis-challenged Ross 708 broilers in response to the *in ovo* injection of 25-hydroxyvitamin D_3_. Animals (Basel).

[bib0020] Fatemi S.A., Williams C.J., Deines J., Peebles E.D. (2023). Vitamin compatibility with the Marek's disease vaccine. Poultry.

[bib0021] Fatemi S.A., Lindsey L.L., Evans J.D., Elliott K.E.C., Leigh S.A., Robinson K.J., Mousstaaid A., Gerard P.D., Peebles E.D. (2023). Effects of the *in ovo* injection of an Escherichia coli vaccine on the hatchability and quality characteristics of commercial layer hatchlings. Poult. Sci..

[bib0022] Fatemi S.A., Mousstaaid A., Williams C.J., Deines J., Poudel S., Poudel I., Elliott K.E.C., Walters E.R., Forcier N., Peebles E.D. (2024). *In ovo* administration of the Marek's Disease vaccine in conjunction with 25-hydroxyvitamin D_3_ and its subsequent effects on the performance and immunity-related characteristics of Ross 708 broiler hatchlings. Poult. Sci..

[bib0023] Fatemi S.A.A.M., Williams C.J., Deines J., Poudel S., Poudel I., Walters E.R., Levy A.W., Peebles E.D. (2024). Effects of the *in ovo* administration of the Marek's Disease Vaccine alone or in combination with the supplemental *in ovo* and dietary administration of 25-hydroxyvitamin D_3_ on the performance, meat yield, and incidence of woody breast myopathy in Ross 708 broilers. Animals (Basel).

[bib0024] Fatemi S.A., Levy A.W., Peebles E.D. (2024). Ross 708 broiler small intestine morphology and immunity improvements in response to *in ovo* Marek's Disease vaccine administration alone or in conjunction with *in ovo* and dietary supplemental cholecalciferol. Poult. Sci..

[bib0025] Fritts C.A., Waldroup P.W. (2003). Effect of source and level of vitamin D on live performance and bone development in growing broilers. J. Appl. Poult. Res..

[bib0026] Genxi Z., Ying T., Tao Z., Jinyu W., Yongjuan W. (2014). Expression profiles and association analysis with growth traits of the MyoG and Myf5 genes in the Jinghai yellow chicken. Mol. Biol. Rep..

[bib0027] Gimeno I.M., Faiz N.M., Cortes A.L., Barbosa T., Villalobos T., Pandiri A.R (2015). *In ovo* vaccination with turkey herpesvirus hastens maturation of chicken embryo immune responses in specific-pathogen-free chickens. Avian Dis.

[bib0028] Gimeno I.M., Glaize A., Cortes A.L. (2018). Effect of Marek's disease vaccines on interferon and toll like receptors when administered *in ovo*. Vet. Immunol. Immunopathol..

[bib0029] Haldar M., Karan G., Tvrdik P., Capecchi M.R. (2008). Two cell lineages, Myf5 and Myf5-independent, participate in mouse skeletall myogenesis. Dev. Cell..

[bib0030] Hutton K.C., Vaughn M.A., Litta G., Turner B.J., Starkey J.D. (2014). Effect of vitamin D status improvement with 25-hydroxycholecalciferol on skeletal muscle growth characteristics and satellite cell activity in broiler chickens. J. Anim. Sci..

[bib0032] Karpala A.J., Lowenthal J.W., Bean A.G. (2008). Activation of the TLR3 pathway regulates IFNbeta production in chickens. Dev. Comp. Immunol..

[bib0033] Karpala A.J., Bagnaud-Baule A., Goossens K.E., Lowenthal J.W., Bean A.G. (2012). Ontogeny of the interferon system in chickens. J. Reprod. Immunol..

[bib0034] Kuttappan V.A., Brewer V.B., Mauromoustakos A., Mc Kee S.R., Emmert J.L., Meullenet J.F., Owens C.M. (2013). Estimation of factors associated with the occurrence of WS in broiler breast fillets. Poult. Sci..

[bib0035] Layton D.S., K.Mara M.D., Malaver-Ortega L.F., Gough T.J., Bruce K., Jenkins K.A., Bean A.G.D. (2022). Interferon signaling in chickens plays a crucial role in inhibiting influenza replication in DF1 cells. Microorganisms.

[bib0036] Lindsey L.L., Elliott K.E.C., Fatemi S.A., Gerard P.D., Peebles E.D. (2023). Utilizing *in ovo* telemetry to examine the effects of reduced incubation temperature on broiler embryo temperature and subsequent hatchability. Poult. Sci..

[bib0037] Liu Q.C., Zha X.H., Faralli H., Yin H., Louis-Jeune C., Perdiguero E., Muñoz-Cànoves P., Rudnicki M.A., Brand M., Perez-Iratxeta C., Dilworth F.J. (2012). Comparative expression profiling identifies differential roles for MyoGenin and p38a MAPK signaling in myogenesis. J. Mol. Cell. Biol..

[bib0038] Lowenthal J.W., Lambrecht B., van den Berg T.P., Andrew M.E., Strom A.D., Bean A.G. (2000). Avian cytokines - the natural approach to therapeutics. Dev. Comp. Immunol..

[bib0039] Mok G.F., Mohammed R.H., Sweetman D. (2015). Expression of myogenic regulatory factors in chicken embryos during somite and limb development. J. Anat..

[bib0040] Moncaut N., Rigby P.W., Carvajal J.J. (2013). Dial M(RF) for myogenesis. FEBS. J..

[bib0041] Morris A., Selvaraj R.K. (2014). *In vitro* 25-hydroxycholecalciferol treatment of lipopolysaccharide-stimulated chicken macrophages increases nitric oxide production and mRNA of interleukin-1 beta and 10. Vet. Immunol. Immunopathol..

[bib0042] Morris A., Shanmugasundaram. M. S. Lilburn R., Selvaraj R.K. (2014). 25-Hydroxycholecalciferol supplementation improves growth performance and decreases inflammation during an experimental lipopolysaccharide injection. Poult. Sci..

[bib0043] Morris A., Shanmugasundaram R., McDonald J., Selvaraj R.K. (2015). Effect of *in vitro* and *in vivo* 25-hydroxyvitamin D treatment on macrophages, T cells, and layer chickens during a coccidia challenge. Anim. Sci. J..

[bib0044] Mousstaaid A., Fatemi S.A., Elliott K.E.C., Levy A.W., Miller W.W., Gerard P.D., Alqhtani A.H., Peebles E.D. (2022). Effects of the *in ovo* and dietary supplementation of L-ascorbic acid on the growth performance, inflammatory response, and eye L-ascorbic acid concentrations in Ross 708 broiler chickens. Animals (Basel).

[bib0045] Mousstaaid A., Fatemi S.A., Elliott K.E.C., Levy A.W., Miller W.W., Olanrewaju H.A., Purswell J.L., Gerard P.D., Peebles E.D. (2023). Effects of the *in ovo* administration of L-ascorbic acid on the performance and incidence of corneal erosion in Ross 708 broilers subjected to elevated levels of atmospheric ammonia. Animals (Basel).

[bib0046] Nong K., Liu Y., Fang X., Qin X., Liu Z., Zhang H. (2023). Effects of the vitamin D_3_ on alleviating the oxidative stress induced by Diquat in Wenchang chickens. Animals (Basel).

[bib0047] Peebles E.D., Barbosa T.M., Cummings T.S., Dickson J., Womack S.K., Gerard P.D. (2017). Comparative effects of *in ovo* versus subcutaneous administration of the Marek's disease vaccine and pre-placement holding time on the processing yield of Ross 708 broilers. Poult. Sci..

[bib72] Peebles E.D. (2018). *In ovo* applications in poultry: a review. Poult. Sci..

[bib0048] Peebles E.D., Barbosa T.M., Cummings T.S., Gerard P.D., Williams C.J., F D.W. (2019). Comparative effects of *in ovo* versus subcutaneous administration of the Marek's disease vaccine and pre-placement holding time on the intestinal villus to crypt ratios of Ross 708 broilers during early post-hatch development. Poult. Sci..

[bib0031] SAS Institute (2013).

[bib0049] Shanmugasundaram R., Selvaraj R.K. (2011). Regulatory T cell properties of chicken CD4+ CD25+ Cells. J. Immunol..

[bib0050] Shanmugasundaram R., Selvaraj R.K. (2012). Vitamin D-1alpha-hydroxylase and vitamin D-24-hydroxylase mRNA studies in chickens. Poult. Sci..

[bib0051] Shanmugasundaram R., Morris A., Selvaraj R.K. (2019). Effect of 25-hydroxy cholecalciferol supplementation on turkey performance and immune cell parameters in a coccidial infection model. Poult. Sci.

[bib0052] Shinin V., Gayraud-Morel B., Gomes D., Tajbakhsh S. (2006). Asymmetric division and cosegregation of template DNA strands in adult muscle satellite cells. Nat. Cell. Biol..

[bib0053] Shojadoost B., Behboudi S., Villanueva A.I., Brisbin J.T., Ashkar A.A., Sharif S. (2015). Vitamin D_3_ modulates the function of chicken macrophages. Res. Vet. Sci..

[bib0054] Sihvo H.K., Immonen K., Puolanne E. (2014). Myodegeneration with fibrosis and regeneration in the pectoralis major muscle of broilers. Vet. Pathol..

[bib0055] Soares J.H., Kerr J.M., Gray R.W. (1995). 25-hydroxycholecalciferol in poultry nutrition. Poult. Sci..

[bib0056] Sojka D.K., Huang Y.H., Fowell D.J. (2008). Mechanisms of regulatory T-cell suppression—a diverse arsenal for a moving target. Immunology.

[bib0057] Steel R.G.D., Torrie J.H. (1980).

[bib0058] Surai P.F., Kochish I.I., Fisinin V.I., Kidd M.T. (2019). Antioxidant defence systems and oxidative stress in poultry biology: An update. Antioxidants (Basel).

[bib0059] Tang D., Wu J., Jiao H., Wang X., Zhao J., Lin H. (2019). The development of antioxidant system in the intestinal tract of broiler chickens. Poult. Sci..

[bib0060] Trocino A., Piccirillo A., Birolo M., Radaelli G., Bertotto D., Filiou E., Petracci M., Xiccato G. (2015). Effect of genotype, gender and feed restriction on growth, meat quality and the occurrence of white striping and wooden breast in broiler chickens. Poult. Sci..

[bib0061] van der Meijden K., Bravenboer N., Dirks N.F., Heijboer A.C., den Heijer M., de Wit G.M.J., Offringa C., Lips P., Jaspers R.T. (2016). Effects of 1,25(OH)_2_ D_3_ and 25(OH)D_3_ on C2C12 myoblast proliferation, differentiation, and myotube hypertrophy. J. Cell. Physiol..

[bib0062] Vignale K., Greene E.S., Caldas J.V., England J., Boonsinchai N., Sodsee P., Pollock E.D., Dridi S., Coon C.N. (2015). 25-Hydroxycholecalciferol enhances male broiler breast meat yield through the mTOR pathway. J. Nutr..

[bib0063] Wan Y.Y., Richard A.F. (2007). Yin-Yang’ functions of transforming growth factor-beta and T regulatory cells in immune regulation. Immunol. Rev..

[bib0064] Wigley P., Kaiser P. (2003). Avian cytokines in health and disease. Braz. J. Poult. Sci..

[bib0065] Williams C.J. (2007). *In ovo* vaccination for disease prevention. Int. Poult. Prod..

[bib0066] Williams C.J. (2011). *In ovo* vaccination and chick quality. Int. Hatch. Prac..

[bib0067] Yablonka-Reuveni Z., Paterson B.M. (2001). MyoD and myogenin expression patterns in cultures of fetal and adult chicken myoblasts. J. Mol. Histol..

[bib0068] Yarger J.G., Saunders C.A., McNaughton J.L., Quarles C.L., Hollis B.W., Gray R.W. (1995). Comparison of dietary 25-hydroxycholecalciferol and cholecalciferol in broiler chickens. Poult. Sci..

[bib70] Yin H., Zhang S., Gilbert E.R., Siegel P.B., Zhu Q., Wong E.A. (2014). Expression profiles of muscle genes in postnatal skeletal muscle in lines of chickens divergently selected for high and low body weight. Poult. Sci..

[bib0069] Zhang L., Tai Y.T., Ho M.Z.G., Qiu L., Anderson K.C. (2017). Interferon-alpha-based immunotherapies in the treatment of B cell-derived hematologic neoplasms in today's treat-to-target era. Exp. Hematol. Oncol..

